# Psychological flexibility in everyday life during post-surgical recovery in youths undergoing spinal fusion surgery and the association with parental responses—a prospective daily diary study using a single-case approach

**DOI:** 10.3389/fpsyg.2025.1610935

**Published:** 2025-10-03

**Authors:** Jenny Thorsell Cederberg, Felicia Sundström, Amani Lavefjord, Sara Laureen Bartels, Rikard K. Wicksell, Lance McCracken, Liesbet Goubert

**Affiliations:** ^1^Department of Psychology, Uppsala University, Uppsala, Sweden; ^2^Department of Clinical Neuroscience, Karolinska Institutet, Stockholm, Sweden; ^3^Department of Psychiatry and Neuropsychology, Maastricht University, Maastricht, Netherlands; ^4^Pain Clinic, Capio St. Göran Hospital, Stockholm, Sweden; ^5^Department of Experimental, Clinical and Health Psychology, Ghent University, Ghent, Belgium

**Keywords:** chronic post-surgical pain, adolescents, recovery, predictors, psychological flexibility, everyday-life, diary, parental factors

## Abstract

**Background:**

Chronic post-surgical pain (CPSP) affects ≈20% of children after major surgery, and the condition is associated with functional disability and ill-health. Psychological flexibility (PF) and parental factors have been shown to predict CPSP in youth following spinal fusion surgery. However, the daily dynamics of these processes throughout post-surgical recovery remain unknown. This study aimed at exploring how PF fluctuates in everyday life for youths undergoing spinal fusion surgery, and to investigate the associations between parental responses and adolescent PF.

**Methods:**

Adolescents with Idiopathic Scoliosis (AIS), aged 12–18 years, undergoing spinal fusion surgery at four hospitals in Belgium, and their parents, completed diaries, measuring adolescent PF and parental responses (including instructions to avoid or engage in activities, parental protective behavior, and parental pain catastrophizing) for 7 days, at five phases: before surgery (T0), at 3 (T1) and 6 weeks (T2), and 6 (T3) and 12 (T4) months, post-surgery. A single-case approach with aggregated results was used, including Tau-U calculations and cross-lagged correlations.

**Results:**

In total, data from 47 adolescents and seven parents were analyzed. Substantial within- and between-person variability characterized the patterns of adolescent PF. Cross-lagged correlations showed bidirectional relationships, demonstrating that parental responses predicted adolescent PF, and that adolescent PF, similarly, predicted parental responses.

**Discussion:**

The results reveal the complex dynamics of PF among adolescents following surgery, and that parent-adolescent patterns after surgery may vary across both individuals and time. These findings also emphasize the need for idiographic pain research and individual-level assessments as well as person-centered treatments in clinical practice.

## Introduction

Chronic post-surgical pain (CPSP) affects around 20% of children and adolescents undergoing major surgery (Rabbitts et al., 2017). The condition is associated with reduced quality-of-life and impaired daily functioning (Dugan et al., 2022) and has been stressed as a public health priority (Furuta et al., 2022; Lo et al., 2022). Spinal fusion surgery is one of the most invasive procedures used for correction of adolescent idiopathic scoliosis (AIS), thus increasing the risk of CPSP and associated long-term health problems for these patients (Connelly et al., 2014; Landman et al., 2011; Wong et al., 2007). Psychological mechanisms have been identified as predictors of pediatric post-surgical recovery, where both child and parental variables are shown to affect child outcomes (Kerr et al., 2024; Rabbitts et al., 2020; Thorsell Cederberg et al., 2024). Anxiety and pain catastrophizing, in both children and parents, are known risk factors for the development and maintenance of CPSP (Chieng et al., 2014; Kerr et al., 2024; Rabbitts et al., 2017; Thorsell Cederberg et al., 2024). Pain catastrophizing is conceptualized as the tendency to magnify the threat value of the pain stimulus, to feel helpless in the context of pain, and by a relative inability to inhibit pain-related thoughts before, during, or following a painful event (Petrini and Arendt-Nielsen, 2020; Quartana et al., 2009). In addition to risk factors, the importance of resilience factors for functioning and well-being in pediatric CPSP is highlighted (Rabbitts et al., 2020). Resilience is defined as adaptive functioning in the presence of stressful circumstances and/or internal distress (Goubert and Trompetter, 2017; Sturgeon and Zautra, 2010). Psychological flexibility (PF), as a resilience factor, has been thoroughly evaluated in chronic pain research (Hayes et al., 2006; Linton, 2005; McCracken et al., 2022). PF is conceptualized as the ability to remain in contact with the present moment, in the presence (or absence) of unpleasant experiences, and to alter or persist in behavior to meet valued ends (Cherry et al., 2021; Hayes et al., 2006). For children with chronic pain, both child and parental PF has been shown to predict improved psychosocial functioning, less negative affect and lower levels of disability (Beeckman et al., 2019; McCracken and Gauntlett-Gilbert, 2011; Timmers et al., 2019). Aside from PF, parental emotional states and behavior are also known to affect child outcomes in pediatric chronic pain (Donnelly et al., 2020). In pediatric CPSP specifically, psychological flexibility in both adolescents and their parents before surgery, and in the adolescents post-surgery, have recently been found to predict adolescent recovery outcomes up to 12 months following spinal fusion surgery (Beeckman et al., 2021; Thorsell Cederberg et al., 2024). Furthermore, in a recent network analysis (Thorsell Cederberg et al., 2025a) investigating associations of adolescent and parental risk and resilience factors in everyday life in pediatric CPSP, PF in adolescents was found to be associated with activity engagement, and parental pain catastrophizing was associated with parent-to-child instructions to avoid activities. These findings further imply the relevance of PF and parental responses in the context of pediatric CPSP.

Although psychological treatments have been shown to reduce pain interference and disability, and improve quality of life, for persons suffering from pain, effect sizes range from small to medium and there is much room for treatment improvement (McCracken et al., 2022). To achieve this, the importance of understanding the needs of the individual has been emphasized, which requires a more idiographic approach, as opposed to traditional nomothetic research, with greater focus on process-based research and treatments (Hayes et al., 2019; McCracken et al., 2022). Intensive longitudinal research, with frequent assessments using quantitative daily diaries, enable in-depth investigations of specific processes, and their dynamic interrelationships in everyday life, and complements group-level analyses with data from a few different time-points (Bolger and Laurenceau, 2013). Diary methods have been described as particularly useful when studying change processes during major events and transitions (Bolger et al., 2003), such as post-surgical recovery. Previous research with adults undergoing major surgery has shown that daily post-surgical catastrophizing and daily emotional distress were associated with pain intensity and pain interference during recovery (Giusti et al., 2022). Similarly, in a study on pediatric chronic pain, diary methods were used to investigate daily antecedents and consequences of pain-related activity avoidance and engagement (Beeckman et al., 2020). It was found that psychological flexibility was associated with lower activity avoidance later in the day and moderated the negative association between pain intensity and activity engagement. In the context of pediatric CPSP, no prior study has, to our knowledge, previously investigated psychological predictors of CPSP using daily measurement and individual-level analyses. Given the empirical evidence of psychological flexibility as a predictor of pediatric pain, and the scientific call for more idiographic, process-based research, a natural next step is to investigate the daily, individual-specific, dynamics of psychological flexibility throughout the post-surgical recovery process. Moreover, it is also crucial to consider the influence of parental factors on pediatric chronic post-surgical pain (CPSP). Specifically, understanding *how* parental emotional responses and behaviors in daily life are associated with adolescents’ psychological flexibility may enhance our knowledge of the psychological predictors of CPSP. This insight may also inform future pain management strategies for children and adolescents undergoing major surgeries. Thus, the aim of the present study was twofold: (1) to explore the fluctuations of daily psychological flexibility in adolescents throughout the peri- and post-surgical recovery process, and, (2) to investigate the association between parental factors and adolescent psychological flexibility in everyday life, for adolescents undergoing spinal fusion surgery.

## Materials and methods

### Design

The study had a longitudinal observational design, using a single-case aggregated approach, with participants completing digital diaries for seven consecutive days, once daily, across five phases pre-determined in length.

### Participants

Patients with Adolescent Idiopathic Scoliosis (AIS), aged 12–18 years, who were scheduled for spinal fusion surgery at four hospitals in Belgium (three university, one general; UZ Ghent, UZ Antwerp, UZ Leuven and Saint-Jan hospital in Bruges), and one of their parents, were offered participation in the study. Recruitment took place at the orthopedic units at the hospitals between 2016 and 2018. Insufficient language skills in Dutch, prior spinal fusion surgery and severe comorbidity due to neurological, developmental, or other health conditions, constituted exclusion criteria. The study is part of the larger research project “Post-operative recovery after spinal fusion (PR-SF) – A prospective study in adolescents with idiopathic scoliosis and their parents,” for which the full protocol can be found at: http://biblio.ugent.be/publication/8578153. The study used a sub-sample of the larger research project (analyzing questionnaire data), in which 100 adolescents and 100 parents were enrolled. The data used in the study are available upon reasonable request. In the present study, 44 adolescents were included in the analyses exploring fluctuations of daily psychological flexibility throughout the post-surgical process, and seven adolescent-parent dyads with sufficient diary data were included in the analyses investigating the association between parental responses and adolescent psychological flexibility. Details of the participant flow are described under “Data preparation” for the general procedure and under “Statistical analyses” for the two separate analyses. Informed consent was obtained from both adolescents and parents. Participants were offered two movie tickets on participation of the study.

### Procedure

The adolescents underwent the standard surgical procedure at their respective hospital. Data were collected through a secure online survey platform (Limesurvey 2.0, Limesurvey Project Team, 2015) where participants completed daily assessments over a period of 1 week at five time-points: 2 weeks before surgery (T0), at 3 (T1) and 6 weeks’ (T2), and 6 (T3) and 12 (T4) months’ post-surgery. Thus, each participant was expected to complete up to 35 diary entries. For each adolescent, the same parent participated throughout the study. If participants did not have access to a computer and/or internet, they were offered to complete the assessments on paper versions. However, no participant chose this alternative. Participants were informed of an upcoming assessment period via telephone 1 week before and prompted to fill in the diary in the evening during seven consecutive days, preferably starting on Mondays. At the beginning of each diary assessment they were asked to complete all items at once. Daily reminders were sent out via automatic text messages. In cases of non-response, participants were reminded by a research team member the following day and asked to complete the diary assessment by 10 a.m that day. Biomedical data was collected from medical records, while demographical data was collected through self-report questionnaires at baseline. The study was approved by the Medical Ethical Committee of Ghent University [BC-2016/0818], with an extended approval from all local ethical committees regarding data collection at their respective unit. The study has adhered to the guidelines for Good Clinical Practice (ICH/GCP) and the Helsinki declaration.

### Diary measures

The diary measures included in the present study were developed based on validated questionnaires measuring constructs of relevance for adolescent pain and functioning in the presence of pain, and applying the Discriminant Content Validity (DCV) procedure developed by Johnston and colleagues (Johnston et al., 2014). Daily psychological flexibility in the adolescents was measured by the following six diary items:” *Today, I was aware of and attentive to my feelings and thoughts”;” Today, I was aware of and attentive to what happened around me”;” Today, I allowed my negative feelings and thoughts to be there”;” Today, I was able to let go of my negative feelings and thoughts”;” Today, I did things which I find important”;” Today, I’m satisfied with the things I have done.”* These diary items were based on the Avoidance and Fusion Questionnaire for Youth (AFQ-Y) (Greco et al., 2008; Livheim et al., 2016) and the DNA-V model from the Thriving Adolescent, an Acceptance and Commitment Therapy and Positive Psychology program, for emotion regulation in teens (Hayes and Ciarrochi, 2015). The six items target awareness of internal and external contingencies (2 items), openness towards feelings and thoughts (2), and engagement in valued activities (2). Parental responses, as reported by parents, were measured using two items on instructions to avoid or engage in activities, two items on parental protective behavior, and three items on parental pain catastrophizing. The avoid/engage instruction items were:” *Today, I told my child to stop or cancel activities because of the pain or other physical complaints”* and*” Today, I told my child to keep on doing fun or important activities (or any other activities that he/she usually does) while he/she was in pain or had other physical complaints.”* These items were derived from the activity avoidance items from the Avoidance Subscale of the Fear of Pain Questionnaire (FOPQ) (Simons et al., 2011) and the Activity Engagement Subscale of the Chronic Pain Acceptance Questionnaire for Adolescents (CPAQ-A) (McCracken et al., 2009), adjusted to parent instructions. The parent protective behavior items were:” *Today, I have made sure that my child did not have to do certain activities (*e.g.*, household chores, going to school or sports) because of the pain or other physical complaints”* and *“Today, I cancelled my activities (job-related duties, household chores and/or hobbies) to be with my child,”* developed from the Solicitous Behavior Subscale of the Inventory of Parent/caregiver Responses to the Children’s Pain Experiences (IRPEDNA) (Huguet et al., 2008). Parental pain catastrophizing was measured by three items derived from the Pain Catastrophizing Scale for Parents (PCS-P) (Goubert et al., 2006):” *Today, I thought something serious might happen to my child because of the pain*”;” *Today, I kept thinking about how much pain my child was experiencing*,” and” *Today, I felt I could not go on much longer because of my child’s pain*.”

All diary items were rated on a 7-point Likert scale ranging from “0” to “6,” from “not at all true” to “totally true.” The psychometric properties of the diary measure have been evaluated and supported (Thorsell Cederberg et al., 2025b).

### Data preparation

In accordance with the guidelines for managing missing momentary data, the cut-off of 33% was applied, meaning that participants who had filled in less than 12 assessments were directly excluded from further analyses (Delespaul, 1995). This concerned seven adolescents (and corresponding parents). Diary entries were coded based on time-point (T0-T4) and day (1–7). Diary entries deviating from this expected pattern were coded based on the instructions to the participants: (1) missing days were regarded as missing data whereby the next diary entry was coded according to the next expected diary day, and (2) a data entry that was registered early the day after was coded as an entry from the previous day. Some participants filled in double assessments the same day, of which only one was kept for analysis (and the other excluded). The rule was to keep the first and delete the second one, under the condition that they contained equal amount of data. If the second one contained more data, this was prioritized and kept, and the first excluded. For diaries exceeding 7 days (longer than the instructed 1-week diary), the seven consecutive days with most data entries were kept, and abundant data entries were disregarded. This procedure was applied for 25 participants. The seven consecutive data entries closest to the starting point were prioritized, in order to correspond best with the relevant time-point.

### Statistical analyses

For the first aim of the study, to explore the fluctuations of daily psychological flexibility in the adolescents throughout the peri- and post-surgical process, a single-case approach was used, with additional aggregated results. For visual analysis, different individual graphs were generated to represent the overall sample. For statistical analysis, non-overlap statistics were used to test for differences between phases, more specifically, Tau-U. As to the second aim of the study, to investigate the temporal association between parental responses and adolescent psychological flexibility on a daily basis, cross-lagged correlations were used to see how changes in the predictor variables were associated with changes in the outcome variables.

### Daily psychological flexibility in adolescents

First, participants responding to less than four assessment points during any of the study phases were excluded from analysis as responding to less than half of the measurement times in a phase might lead to difficulties capturing the variation across time for that individual. From the original 93 adolescents (i.e., remaining after the first 33% cut-off exclusion), 44 participants remained after this exclusion, which is still a large number for a single case design study. To address remaining missing data, a function within the scan package was used (Wilbert and Lueke, 2023) to interpolate missing data, as this has shown to lead to more valid results (Wilbert, 2023). Inspections of the data indicated potential trend effects, meaning that scores might change over time itself rather than just across phases, meaning that any statistical methods employed needed to take this into consideration. Changes across phases were therefore analyzed using the non-parametric Tau-U statistic (suitable for individual-level analyses), a statistic able to control for trends in the data (Parker et al., 2011), not particularly affected by temporal dependency (Brossart et al., 2018) providing an effect size as well as a *p*-value for hypothesis testing (Manolov and Moeyaert, 2017). Originally, Kendall (1938) introduced the Tau statistic as a rank correlation statistic, with values of +1 indicating a perfect positive relationship, values of −1 indicating a perfect negative relationship, and 0 indicating no relationship. As Tau is calculated by comparing each score to every future score, and determining whether the future score is higher, lower or the same as the earlier score, an adjusted formula was developed (Brossart et al., 2018) for whenever ties were present, meaning that one or more future score(s) was the same as an earlier one.

In a single case design, scores within a first phase are set to represent one time-point, whereas scores within a second phase are set to represent another time-point (Brossart et al., 2018). When time is converted to phase variables in this way, trend information is lost, which is important to understand as it might confound results seen across phases. Therefore, Tau-U, an extension of Tau, was used, merging methods from Tau with methods from Mann–Whitney U (Parker et al., 2011), where pairwise comparisons are conducted both within a particular phase and between phases. One can report on Tau-U controlling for trend(s) in one or both phases (Brossart et al., 2018), leading to different Tau-U statistics often being called the same thing, stressing the importance of defining which statistic is reported. Furthermore, there are some Tau-U formulas available that one need to be aware about as they may inflate results and cause values to fall outside the bounds of ±1 (Brossart et al., 2018).

In this study, the default settings in the scan package were used to calculate Tau-U, using a formula adjusting for ties (“Kendall’s Tau B”), and a formula not leading to inflated results that may fall outside the ±1 bounds (method set to “complete”) (Wilbert and Lueke, 2023). Results were thus based on the conservative Tau-U_A vs. B + Trend B - Trend A_ to control for trends within phases while comparisons were made between phases. Multiple calculations were conducted to test changes across phases following each other and between the baseline phase 0 and the later phases. A meta-analysis of the Tau-U for all included participants was also included, using the scan package’s default setting of weighing averages using *z*-scores (Wilbert and Lueke, 2023).

### Association between daily parental responses and adolescent psychological flexibility

In order to assess the temporal direction of correlations between proposed processes and outcome variables, cross-lagged correlations adjusted for autocorrelations were used (Borckardt et al., 2008; Caneiro et al., 2019). This provides a temporal association between the variables and the possibility to find significant associations at several time-points, either before (negative lag), during (zero lag), or after the outcome (positive lag).

Non-parametric cross-lagged correlations using lags −2 to +2 were conducted using Simulation Modeling Analysis (SMA) (Borckardt et al., 2008). SMA is software designed for short time series, commonly used in idiographic designs, and enables the researcher to conduct cross-lagged correlations based on their collected data and simulated data. The software creates random streams of data similar to the original data, corresponding to the case where no correlation is present between the two variables, using the same number of observations as the collected data. The software then uses these random streams of data in order to calculate the values of the correlations, comparing the observed correlations in the collected data with the generated streams. This also enables the software to calculate *p*-values relative to the set alpha level. Due to the large number of simulations conducted, Bonferroni corrections were also used (Borckardt et al., 2008).

In the present study, the data was not normally distributed; thus, Spearman rank correlations were used. The number of simulations was set to 5,000 for all analyses, and Bonferroni corrections were employed. This method divides the preset alpha value (*α*; typically, 0.05) by the number of lags plus one (for lag-0), to correct for multiple comparisons. Since the number of cross-lagged correlations calculated was large, a conservative alpha level of 0.01 was set for the present analyses to further avoid Type I errors. If insufficient variation in the data was discovered, analyses could not be performed. Similarly, if too much missing data was present, analyses were not run due to the limited number of observations for each phase.

With regard to interpretation, the program always assumed that parental factors preceded adolescent psychological flexibility. Therefore, positive lags (e.g., Lag+1, Lag+2) indicated that changes in parental responses preceded changes in adolescent psychological flexibility while negative lags (e.g., Lag-1, Lag-2) indicated that changes in adolescent psychological flexibility preceded changes in parental responses. Lag 0 indicated simultaneous changes in adolescent psychological flexibility and parental responses.

Seven adolescent-parent dyads included sufficient data from both adolescent and parent for at least one of the variable pairs and were, therefore, included in the cross-lagged correlational analyses. Four of these adolescents were also included in the Tau-U analyses, and three were not. The parent avoid instruction variable was not included in the analyses due to insufficient data.

## Results

### Sample characteristics

The sample comprised 47 adolescents and seven parents. Sample characteristics for the adolescents are presented in Table 1. Among the parents, five were mothers, one was father, and one did not report. Four parents reported being married or co-habiting, two were divorced, and one did not report marital status. Age of parents ranged from 37 to 55 years.

**Table 1:** Sample characteristics of the adolescents.

Characteristic	Adolescents (*n* = 47)
Age (yrs.) (M (SD))	15.2 (1.6)
Range (min-max)	12-18
Sex (*n* (%))	
Male	9 (19.1)
Female	38 (80.9)
Level of education (*n* (%))	
Primary school	0
High school	47 (100)
Ethnicity (*n* (%))	
Western European	43 (91.4)
Eastern European	1 (2.1)
South-eastern Asian	1 (2.1)
Not reported	2 (4.3)
Biomedical variables (M (SD))	
Height (cm)	165.6 (7.6)
Weight (kg)	55.5 (11.4)
BMI	20.1 (3.9)
Cobb angle (degrees)	53.1 (10.4)
Duration of hospital stay	
<7 days	28 (59.6)
7–14 days	15 (31.9)
15–21 days	1 (2.1)
Not reported	3 (6.4)

### Daily psychological flexibility in adolescents during and after surgery

**Figure 1:**
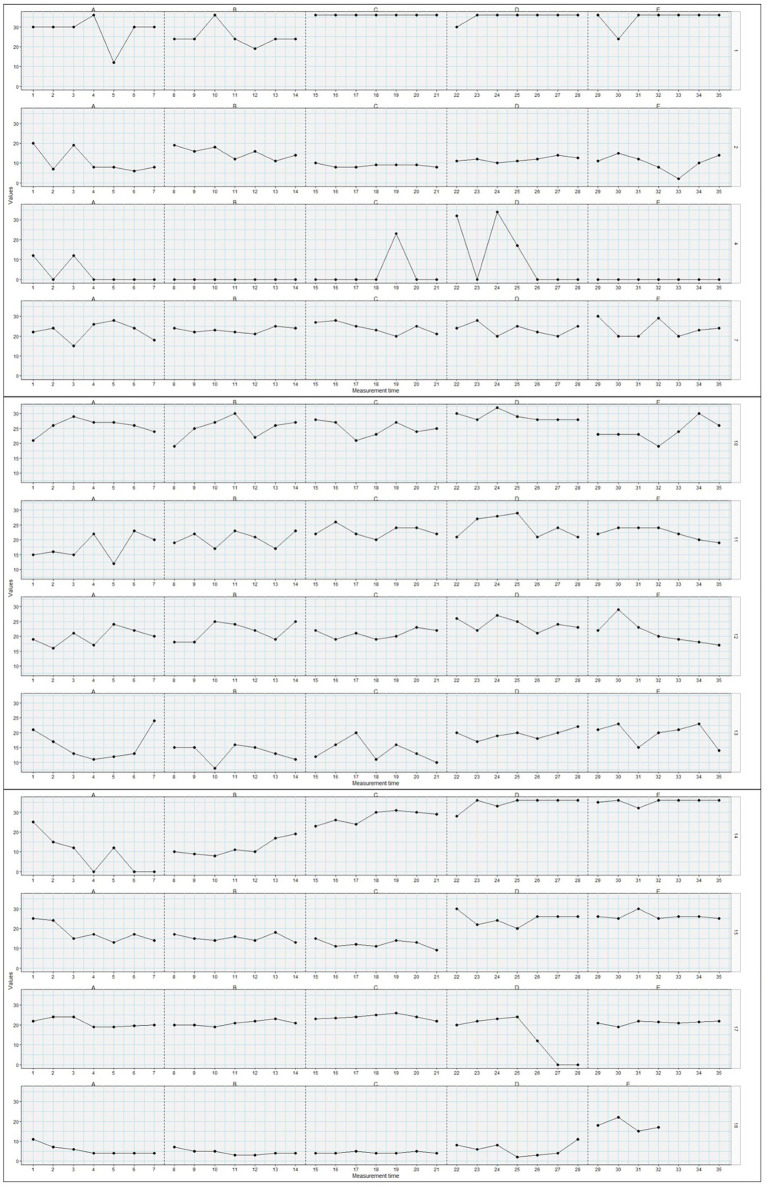
Plots of changes in psychological flexibility (y-axis) throughout the post-surgical phases (T0-T4=A-E; x-axis) for adolescents/participants 1, 2, 4, 7, 10–15, 17, and 18 (*n* = 12).

**Figure 2:**
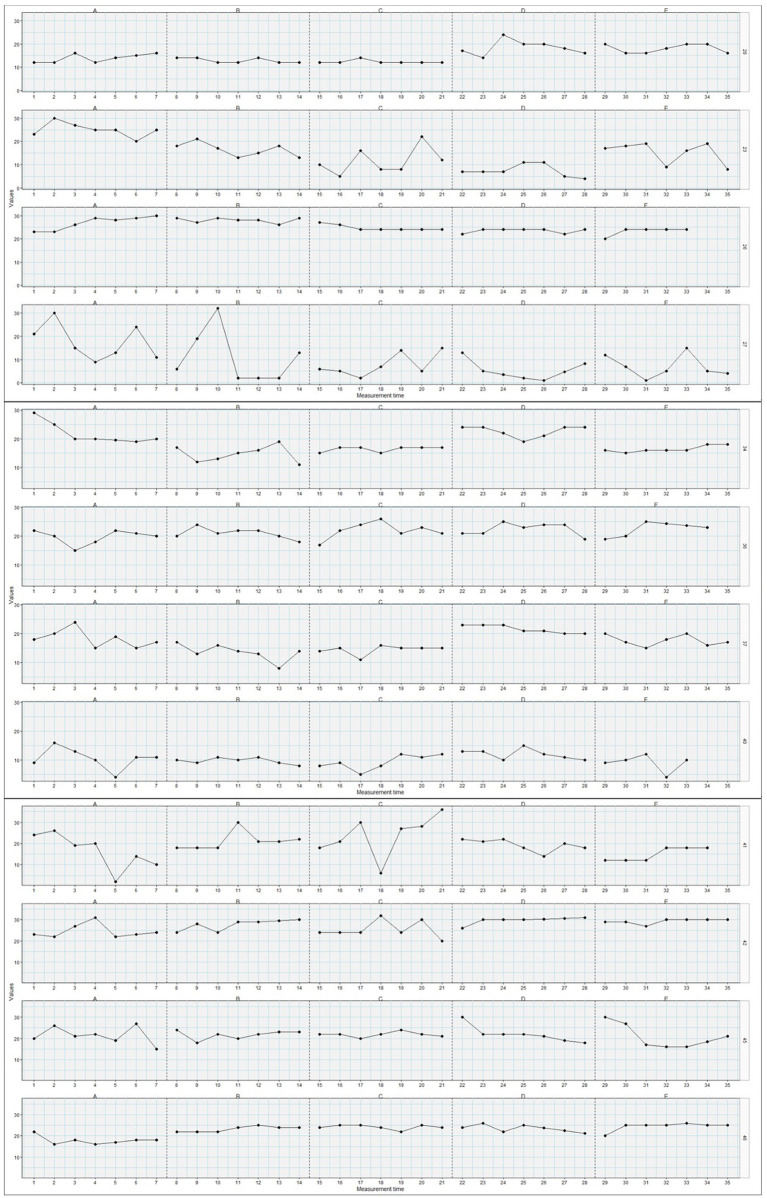
Plots of changes in psychological flexibility (y-axis) throughout the post-surgical phases (T0-T4=A-E; x-axis) for adolescents/participants 20, 23, 26, 27, 34, 36, 37, 40–42, 45, and 46 (*n* = 12).

**Figure 3:**
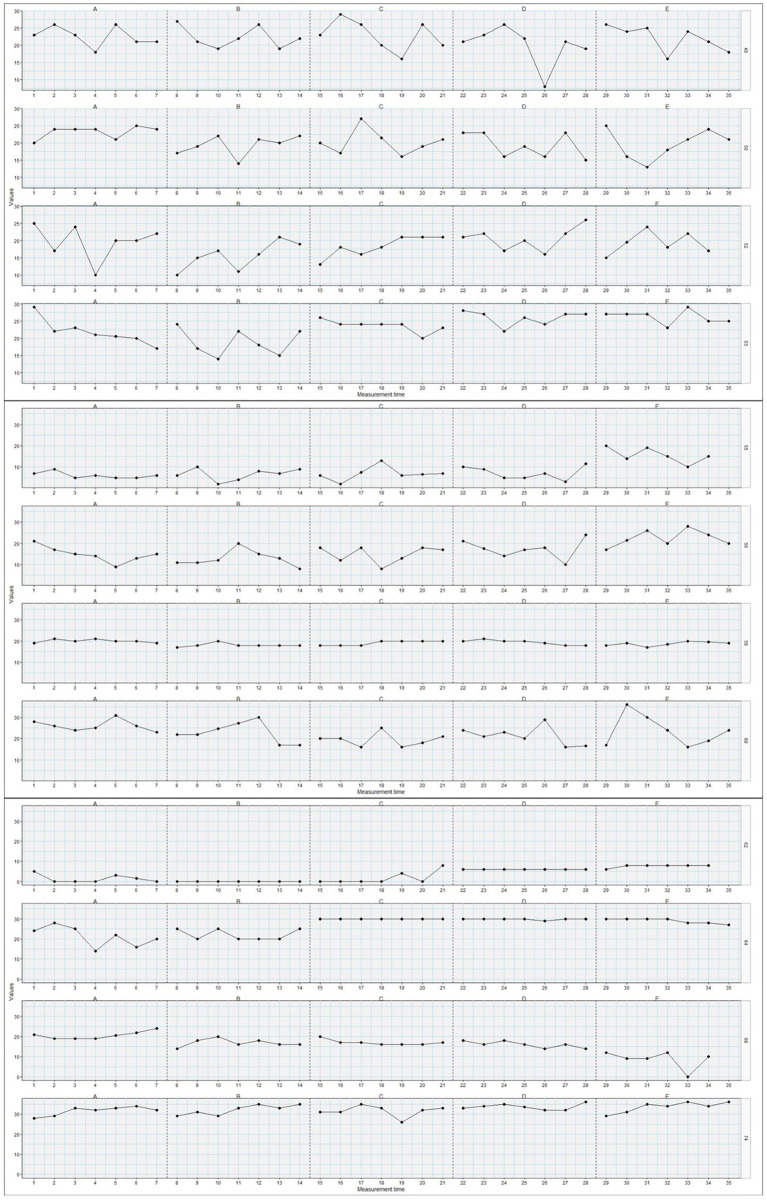
Plots of changes in psychological flexibility (y-axis) throughout the post-surgical phases (T0-T4=A-E; x-axis) for adolescents/participants 49, 50, 52, 53, 55, 56, 58, 60, 62, 64, 68, and 74 (*n* = 12).

**Figure 4:**
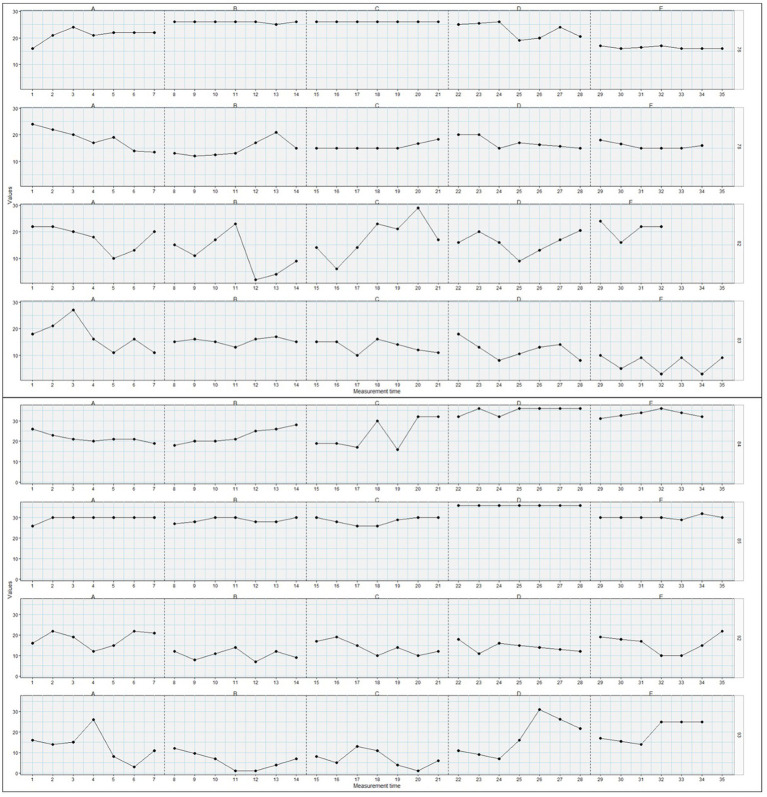
Plots of changes in psychological flexibility (y-axis) throughout the post-surgical phases (T0-T4=A-E; x-axis) for adolescents/participants 76, 78, 82–85, 92, and 93 (*n* = 12).

Detailed results from the Tau-U analyses are presented in Tables 2, 3. Of the 44 adolescents, 11 showed statistically significant changes in psychological flexibility from phase 0 to phase 1, 10 from phase 1 to phase 2, 16 from phase 2 to phase 3, and 11 from phase 3 to phase 4. Comparing the later phases (2–4) to the baseline phase, 12 individuals had statistically significant changes in psychological flexibility from phase 0 to 2, 15 from 0 to 3, and 19 from 0 to 4. Thus, it was most common for change to occur between phase 0, pre-surgery, and one of the later phases, at long term follow-up, but also between phase 2 and 3, at 6 weeks’ to 6 months’ follow-up. There was great variety in the patterns of changes in psychological flexibility between individuals. While some individuals *increased* their psychological flexibility from one phase to another, others *decreased* their psychological flexibility, and the particular phase(s) where changes occurred, differed on an individual basis. Additionally, while some individuals had changes in psychological flexibility only at one particular phase comparison, others displayed changes in many of the phase comparisons. Furthermore, the direction of change within one particular person changed between phase comparisons for some individuals. For instance, individual 2 showed decreases in their levels of psychological flexibility between phase 1 and 2, and then showed increased levels of psychological flexibility between phases 2 and 3. Other individuals displayed more consistent patterns of change across time. While most participants did not display statistically significant changes between phases, on an aggregated level, there were significant changes observed in increased levels of psychological flexibility between phases 0 and 3, between 0 and 4, and between 2 and 3. Inspecting these phase changes, they all showed small positive changes in psychological flexibility. Plots of changes in psychological flexibility throughout the phases (T0–T4), for each adolescent, are presented in Figures 1–4.

**Table 2:** Tau-U results for (adolescent) psychological flexibility for each case as well as aggregated results, between each phase (0–4) and the phase directly following it.

***P***	0 vs 1			1 vs 2			2 vs 3			3 vs 4		
	Tau-U	Var	Z	Tau-U	Var	Z	Tau-U	Var	Z	Tau-U	Var	Z
1	−0.33	17.3	−1.5	**0.65**	**15.86**	2.84**	−0.03	8.06	−0.12	−0.06	11.00	−0.27
2	0.2	18.11	0.99	**−0.46**	**18.04**	−2.22*	**0.72**	**17.98**	3.45**	−0.26	18.01	−1.28
4	−0.13	10.95	−0.55	0.26	8.06	1.12	0.06	14.45	0.28	−0.21	12.99	−0.92
7	−0.05	17.93	−0.22	0.00	18.06	0.00	0.01	17.90	0.06	0.01	17.75	0.06
10	0.07	17.93	0.33	−0.16	18.00	−0.78	**0.47**	**17.78**	2.25*	−0.22	17.90	−1.06
11	0.22	18.08	1.05	0.25	17.94	1.23	0.09	17.96	0.45	−0.29	17.90	−1.40
12	0.18	18.13	0.88	−0.08	18.01	−0.39	0.28	18.08	1.38	**−0.41**	**18.21**	−2.03*
13	−0.16	18.04	−0.78	0.06	18.01	0.28	**0.62**	**18.00**	3.00**	0.01	17.97	0.06
14	0.36	18.11	1.77	**0.52**	**18.21**	2.58*	**0.47**	**17.78**	2.25*	0.00	14.45	0.00
15	−0.08	18.01	−0.39	**−0.42**	**18.08**	−2.05*	**0.64**	**18.14**	3.14**	0.05	17.34	0.23
17	0.21	17.98	1.00	**0.43**	**18.13**	2.10*	**−0.56**	**18.08**	−2.71*	0.26	18.08	1.27
18	−0.11	17.39	−0.52	0.17	16.72	0.78	0.12	17.42	0.57	**0.48**	**12.81**	2.03*
20	**−0.46**	**16.72**	−2.09*	−0.13	14.14	−0.57	**0.58**	**17.42**	2.76*	0.00	17.53	0.00
23	**−0.57**	**18.11**	−2.76*	−0.16	18.18	−0.77	**−0.42**	**18.08**	−2.05*	**0.47**	**18.11**	2.32*
26	−0.17	17.64	−0.79	**−0.64**	**17.62**	−3.01**	−0.17	14.41	−0.76	0.07	10.94	0.27
27	−0.21	18.14	−1.05	0.15	17.97	0.72	−0.37	18.14	−1.82	0.12	18.14	0.61
34	−0.37	18.14	−1.82	0.31	17.37	1.44	**0.51**	**17.53**	2.40*	**−0.43**	**17.76**	−2.03*
36	0.08	17.73	0.39	0.26	18.01	1.28	0.03	17.90	0.17	0.04	16.24	0.18
37	**−0.48**	**18.16**	−2.37*	0.30	17.87	1.45	0.33	17.87	1.57	−0.38	17.87	−1.79
40	−0.22	17.82	−1.07	0.16	17.91	0.78	0.11	18.06	0.55	−0.23	14.21	−0.99
41	**0.42**	**18.14**	2.04*	0.15	17.90	0.73	**−0.47**	**18.11**	−2.32*	−0.14	15.73	−0.64
42	**0.49**	**18.08**	2.38*	−0.39	17.42	−1.84	**0.58**	**17.79**	2.76*	−0.39	16.98	−1.83
45	0.16	18.11	0.77	−0.13	17.39	−0.63	−0.32	16.98	−1.47	−0.06	18.08	−0.28
46	**0.66**	**17.80**	3.15**	0.08	16.97	0.35	−0.26	17.76	−1.24	**0.45**	**17.45**	2.12*
49	0.00	17.98	0.00	0.01	18.08	0.06	−0.15	18.08	−0.72	0.07	18.11	0.33
50	**−0.42**	**17.94**	−2.01*	−0.09	18.13	−0.44	−0.16	18.04	−0.78	0.21	18.04	1.00
52	−0.13	18.18	−0.66	0.26	17.97	1.28	0.09	17.94	0.45	−0.16	16.25	−0.74
53	0.01	18.14	0.06	0.30	17.78	1.46	**0.49**	**17.67**	2.32*	−0.01	17.45	−0.06
55	0.24	18.01	1.17	−0.02	18.11	−0.11	−0.09	18.18	−0.44	**0.48**	**16.30**	2.27*
56	−0.07	18.11	−0.33	0.16	18.06	0.78	0.14	18.00	0.67	0.38	18.18	1.87
58	−0.43	17.50	−2.00	**0.48**	**15.86**	2.08*	−0.27	16.51	−1.21	0.06	17.82	0.28
60	−0.20	18.18	−0.99	−0.24	18.16	−1.16	0.05	18.04	0.22	0.17	18.14	0.83
62	−0.31	12.99	−1.39	**0.48**	**11.00**	2.09*	0.35	16.51	1.57	**0.72**	**13.66**	2.93**
64	0.13	17.56	0.63	**0.68**	**16.64**	3.06**	−0.26	8.06	−1.12	**−0.45**	**14.41**	−2.01*
68	**−0.62**	**18.04**	−2.99**	−0.06	17.31	−0.29	−0.24	17.31	−1.10	**−0.44**	**16.16**	−2.04*
74	0.14	17.87	0.67	−0.19	17.80	−0.90	0.26	18.01	1.28	0.22	18.01	1.06
76	**0.44**	**17.34**	2.08*	0.32	8.06	1.36	**−0.62**	**16.38**	−2.87**	**−0.59**	**18.00**	−2.89**
78	0.01	18.21	0.05	0.21	17.45	0.97	−0.04	16.98	−0.18	−0.07	15.84	−0.32
82	−0.21	18.21	−1.04	0.40	18.18	1.98	−0.18	18.18	−0.88	0.28	12.66	1.18
83	0.01	17.90	0.06	**−0.45**	**17.70**	−2.15*	−0.12	18.16	−0.61	−0.38	18.08	−1.82
84	**0.43**	**17.90**	2.07*	−0.18	18.18	−0.88	**0.56**	**17.53**	2.62*	−0.34	15.35	−1.50
85	−0.23	15.43	−1.04	−0.06	17.19	−0.29	**0.57**	**16.87**	2.61*	**−0.65**	**16.51**	−2.91**
92	**−0.55**	**18.14**	−2.70*	0.20	18.11	0.99	0.06	18.16	0.28	0.22	18.18	1.10
93	−0.37	18.21	−1.81	0.09	18.11	0.44	**0.57**	**18.24**	2.85**	0.07	16.28	0.31
												
All	−0.06	0.05	−1.30	0.09	0.05	1.90	**0.11**	**0.05**	2.40*	−0.04	0.05	−0.82

Reporting 0 vs 1 + Trend 1 – Trend 0. P = Participant; Var = Variability = Standard Deviation of Score differences between phases (SD_S) for individuals and Standard Error of the tau estimate (SE) for aggregated results. Statistically significant results reported in bold; *p < 0.05; **p < 0.01.

**Table 3:** Tau-U results for (adolescent) psychological flexibility for each case as well as aggregated results, between baseline and later phases.

*P*	0 vs. 2			0 vs. 3			0 vs. 4		
	Tau-U	Var	*z*	Tau-U	Var	*z*	Tau-U	Var	*z*
1	**0.62**	**15.86**	2.71*	**0.61**	**16.16**	2.66*	**0.49**	**16.51**	2.18*
2	0.21	17.37	0.98	**0.45**	**18.11**	2.21*	0.15	18.03	0.72
4	0.09	12.95	0.39	0.17	15.51	0.77	−0.13	10.95	−0.55
7	−0.04	18.18	−0.22	0.00	18.06	0.00	0.03	18.06	0.17
10	−0.12	17.94	−0.56	0.37	17.94	1.78	−0.10	18.01	−0.50
11	0.32	17.94	1.56	0.33	18.14	1.60	0.17	18.01	0.83
12	0.10	18.01	0.50	0.27	18.18	1.32	−0.19	18.16	−0.94
13	−0.19	18.08	−0.94	0.36	18.11	1.77	0.25	18.11	1.21
14	**0.79**	**18.11**	3.87**	**0.87**	**17.67**	4.13**	**0.85**	**17.67**	4.02**
15	−0.38	18.13	−1.88	0.59	18.11	2.87**	**0.64**	**17.90**	3.07**
17	**0.47**	**17.97**	2.28*	−0.16	18.06	−0.78	0.19	17.98	0.89
18	0.06	15.51	0.26	0.19	17.72	0.90	**0.79**	**12.50**	3.28**
20	**−0.53**	**15.48**	−2.39*	0.31	18.01	1.50	0.40	17.60	1.88
23	−0.39	18.14	−1.93	**−0.55**	**18.04**	−2.66*	**−0.53**	**18.14**	−2.59*
26	**−0.55**	**17.72**	−2.60*	**−0.52**	**17.72**	−2.48*	**−0.52**	**14.21**	−2.25*
27	−0.23	18.21	−1.15	**−0.46**	**18.24**	−2.30*	**−0.41**	**18.21**	−2.03**
34	−0.35	17.67	−1.64	0.29	17.90	1.40	−0.27	17.90	−1.29
36	0.32	18.04	1.55	0.35	18.08	1.71	0.37	16.25	1.72
37	−0.31	17.47	−1.49	0.26	18.01	1.28	−0.01	17.94	−0.06
40	0.01	18.08	0.06	0.07	17.96	0.33	−0.13	14.35	−0.56
41	**0.54**	**18.27**	2.68*	0.10	18.16	0.50	0.13	16.17	0.62
42	0.11	17.75	0.51	**0.56**	**18.08**	2.71*	**0.48**	**17.92**	2.29**
45	0.13	17.75	0.62	−0.13	17.97	−0.61	−0.11	18.18	−0.55
46	**0.52**	**17.91**	2.46*	0.38	18.11	1.88	**0.63**	**17.67**	3.00**
49	0.00	17.87	0.00	−0.18	17.82	−0.84	−0.09	17.98	−0.44
50	−0.40	17.97	−1.95	**−0.62**	**17.90**	−2.96**	−0.30	17.67	−1.41
52	0.05	18.11	0.22	0.09	18.04	0.44	−0.08	16.27	−0.37
53	0.36	17.97	1.72	**0.55**	**18.14**	2.70*	**0.56**	**18.08**	2.71*
55	0.31	17.90	1.51	0.14	17.72	0.68	**0.57**	**16.22**	2.65*
56	0.17	18.08	0.83	0.35	18.16	1.71	**0.65**	**18.18**	3.19**
58	−0.07	16.84	−0.30	−0.32	17.23	−1.45	−0.26	17.76	−1.24
60	**−0.42**	**18.16**	−2.04*	−0.40	18.18	−1.98	−0.17	18.14	−0.83
62	0.13	15.55	0.58	**0.68**	**16.75**	3.10**	**0.72**	**15.60**	3.21**
64	**0.73**	**17.01**	3.41**	**0.67**	**17.47**	3.20**	**0.46**	**17.93**	2.23*
68	**−0.72**	**17.96**	−3.45**	**−0.82**	**18.01**	−3.94**	**−0.73**	**16.22**	−3.39**
74	−0.10	17.90	−0.50	0.17	17.90	0.84	0.32	18.06	1.55
76	**0.52**	**16.87**	2.37*	−0.02	18.11	−0.11	**−0.67**	**17.64**	−3.17**
78	0.13	17.80	0.62	−0.05	18.11	−0.22	−0.04	16.28	−0.18
82	0.25	18.18	1.21	−0.01	18.08	−0.06	0.47	12.46	1.93
83	−0.25	18.04	−1.22	−0.25	18.13	−1.21	**−0.49**	**18.98**	−2.38*
84	0.18	18.04	0.89	**0.85**	**17.67**	4.02**	**0.79**	**16.25**	3.69**
85	−0.29	15.43	−1.30	**0.61**	**16.16**	2.66*	0.03	12.99	0.15
92	**−0.50**	**18.13**	−2.43*	**−0.48**	**18.16**	−2.37*	−0.24	18.08	−1.16
93	−0.30	18.21	−1.48	0.34	18.21	1.70	**0.50**	**16.25**	2.34*
									
All	0.03	0.05	0.65	**0.16**	**0.05**	3.60**	**0.14**	**0.05**	3.10**

Reporting A vs. B + Trend B - Trend A. P=Participant; Var = Variability = Standard Deviation of Score differences between phases (SD_S) for individuals and Standard Error of the tau estimate (SE) for aggregated results. Statistically significant results reported in bold; *p < 0.05; **p < 0.01.

### Association between parental factors and adolescent psychological flexibility

Cross-lagged correlations including the strongest correlations found at specific lags (positive or negative), with different temporal directions represented for each participant are presented in Tables 4–6.

**TABLE 4:** Cross-lagged correlations between parental protective behavior and adolescent psychological flexibility.

Phase	Participant									
	P10		P14		P24		P85		P17	
Phase 0	0.76*	Lag+2	0.37	Lag-2	#	#	-	-	0.54	Lag+1
Phase 1	0.60	Lag+1	−0.70	Lag-1	#	#	0.69	Lag+1	0.92	Lag+2
Phase 2	-	-	−0.89	Lag-2	0.97	Lag+2	0.54	Lag-1	0.50	Lag-2
Phase 3	-	-	-	-	-	-	-	-	-	-
Phase 4	-	-	−77	Lag-1	-	-	-	-	-	-

The table shows the output from the lag with the strongest correlation; − = insufficient variation; # = missing data; *p = > 0.01; **p > 0.001. Participant refers to adolescent. Yellow cells indicate positive lags; blue cells indicate negative lags.

**TABLE 5:** Cross-lagged correlations between parental engagement instructions and adolescent psychological flexibility.

Phase	Participant									
	P10		P14		P17		P24		P85	
Phase 0	-	-	0.76*	Lag+2	0.46	Lag-2	#	#	-	-
Phase 1	0.67	Lag-2	0.88*	Lag+1	0.43	Lag+1	#	#	0.71	Lag+1
Phase 2	0.87	Lag+2	0.73	Lag+2	0.44	Lag+2	0.76	Lag-2	0.54	Lag-1
Phase 3	0.72	Lag 0	0.45	Lag 0	-	-	-	-	-	-
Phase 4	-	-	-	-	-	-	-	-	-	-

The table shows the output from the lag with the strongest correlation; − = insufficient variation; # = missing data; *p = > 0.01; **p > 0.001. Participant refers to adolescent. Yellow cells indicate positive lags; blue cells indicate negative lags; green cells indicate zero-lags.

**TABLE 6:** Cross-lagged correlations between parental catastrophizing and adolescent psychological flexibility.

Phase	Participant									
	P10		P17		P24		P43		P73	
Phase 0	0.66	Lag 0	−0.46	Lag-2	#	#	0.66	Lag+1	−0.95**	Lag 0
Phase 1	-	-	0.81	Lag+1	#	#	-	-	-	-
Phase 2	0.62	Lag 0	0.67	Lag-1	−0.53	Lag-2	-	-	-	-
Phase 3	0.70	Lag-1	-	-	-	-	-	-	#	#
Phase 4	-	-	-	-	−0.71	Lag-2	#	#	#	#

The table shows the output from the lag with the strongest correlation; − = insufficient variation; # = missing data; * *p* = > 0.01; **p > 0.001. Participant refers to adolescent. Yellow cells indicate positive lags; blue cells indicate negative lags; green cells indicate zero-lags.

Cross-lagged correlations between parental protective behavior and adolescent psychological flexibility (PF) across the different phases for five participants (P10, P14, P24, P85, P17) are presented in Table 4. Parental protective behavior preceded adolescent PF in the model. For participant P10, parental protective behavior preceded changes in psychological flexibility, with a statistically significant correlation in phase 0 (and a non-significant correlation in phase 1). For participant P24 the same pattern, although non-significant, was disclosed in phase 2. For participants P85 and P17, although non-significant, parental protective behavior preceded adolescent PF in phase 0 (only P17) and 1, but vice versa in phase 2, where adolescent PF preceded parental protective behavior. For P14, adolescent PF preceded parental protective behavior in phases 0, 1, 2, 4.

Cross-lagged correlations between parental instructions to engage in activities and adolescent PF across the different phases for five participants (P10, P14, P17, P24, P85) are presented in Table 5. Parental instructions to engage in activities preceded child PF. For participant P14, parental instructions to engage in activities preceded changes in psychological flexibility, with significant correlations in phase 0 and 1. For participants P10, P17, and P85, both positive and negative lags were apparent, indicating, although non-significant, both that parental instructions preceded adolescent PF, and vice versa. For P24, although non-significant, adolescent PF preceded parental instructions to engage in activities in phase 2.

Cross-lagged correlations between parental catastrophizing and adolescent PF across different phases for five participants (P10, P17, P24, P43, P73) are presented in Table 6. Parental catastrophizing preceded child PF. For participant P73, a statistically significant correlation was shown between parental pain catastrophizing and adolescent PF in phase 0. For P17, although non-significant, both positive and negative lags were shown, indicating both parental pain catastrophizing preceding adolescent PF and vice versa.

## Discussion

The present study explored the within- and between-person fluctuations of daily psychological flexibility in adolescents throughout the peri- and post-surgical process, and investigated the association between parental factors and adolescent psychological flexibility in everyday life, for adolescents undergoing spinal fusion surgery.

The results indicated that while many adolescents displayed rather consistent patterns of psychological flexibility throughout the post-surgical recovery process, there were still significant changes observed between phases, where a number of adolescents showed increased psychological flexibility from before surgery to long-term follow-up (at six and 12 months), and from 6 weeks to 6 months after surgery. Furthermore, the results disclosed a great variety of patterns in psychological flexibility fluctuations between individuals. While at an aggregated level, psychological flexibility *increased* from one phase to another, on an individual level, psychological flexibility also *decreased* in some adolescents. The particular phase(s) where changes occurred also differed between individuals. Additionally, for some individuals, the direction of change for that particular person changed between phases. Also, the aggregated results do not represent the individuals displaying larger effects. The non-significant results observed on the aggregated level between phases 0 and 1 (i.e., before surgery and 3 weeks’ post-), 0 and 2 (i.e., before surgery and 6 weeks’ post-), 1 and 2 (i.e., 3 and 6 weeks’ post-surgery), and, 3 and 4 (i.e., 6 and 12 months’ post-surgery), do not reflect that there are particular individuals showing larger effects in these phase changes, in either direction. Overall, the present results illustrate the heterogeneity between individuals and emphasize the need for individual analyses and interventions, in line with the research call for a more idiographic process-based approach in the context of pain (Hayes et al., 2019; McCracken et al., 2022). Concretely, this approach means assessing individuals, such as participants in research or patients in clinic, on a daily or weekly basis to capture the mechanisms at play for a specific person over a specific period of time. These results are particularly important in the light of previous research showing that psychological flexibility before and after surgery predict long-term recovery in adolescents undergoing spinal fusion surgery (Thorsell Cederberg et al., 2024).

Regarding the daily associations between parental factors and adolescent psychological flexibility throughout the post-surgical recovery process, the results showed that for some participants, parental factors predicted changes in psychological flexibility, while for others, the direction was the opposite. The existence of both strong positive and negative correlations, some of which were not statistically significant, suggests that parent–child dynamics are influenced by a multitude of factors that may operate differently across both individuals and time. These results are in line with previous research showing that parental factors are associated with child outcomes (Donnelly et al., 2020; Thorsell Cederberg et al., 2024), but further underscores the importance of personalized or idiographic studies in capturing these unique relationships rather than relying solely on group-level analyses.

While multi-level modeling approaches are possible for this kind of data, with both many participants and many assessment points, for studying the fluctuations over time, we relied on the Tau-U as a standard single case statistical method preserving the individual specific time series rather than aggregating the data in more nomothetically oriented analyses. This in line with recommendations against aggregating data when the aim is individual-level understanding (Lavefjord and Sundström, 2025). Granted, the meta-tau that is included does aggregate the data, but is only used as a small complement alongside the individual analyses, and with the main focus being the individual level analyses, it becomes clear that the aggregated meta-tau is not representative of all participants – insight that might not be as clear with output from more nomothetically oriented analyses. To investigate the association between daily parental factors and adolescent psychological flexibility, a simulation-based cross-lagged correlation analysis using the Simulation Modeling Analysis (SMA) software was chosen for two primary reasons. First, the method is specifically designed to handle the short time-series data (*N* < 30 per phase) that is characteristic of our dataset. Second, while it is not a panel model, the method explicitly accounts for the confounding influence of autocorrelation. It achieves this by generating an empirical *p*-value from thousands of Monte Carlo simulations. These simulations use random data streams that are programmed to have the same lag-1 autocorrelation and N-size as the observed data. This provides a robust test against Type I errors that can arise from serial dependency in short time-series. Furthermore, this analytical approach has previously been used for analyzing time-series data in the field of chronic pain (Caneiro et al., 2019). While a cross-lagged panel model (CLPM) is a powerful alternative, its data requirements are often not met by shorter time-series, making the simulation-based approach a more suitable and pragmatic choice for the present dataset. However, future studies should also explore more advanced statistical tests outside of cross-lagged correlations to investigate these research questions further.

### Limitations

There was a substantial amount of missing data in the study. Although the number of participants was still large for a single case study, and adequate for the analyses conducted, the missing data may affect the generalizability of the results, and led to limited data variation. Also, for the variable parental instructions to avoid activities, the association with adolescent psychological flexibility could not be assessed due to limited data. Missing data was mainly due to insufficient diary data completion across phases combined with data quality requirements (i.e., not recruitment challenges or participant attrition), and particularly concerned parents. One reason for this could be that parents may have underestimated the importance of their contribution of diary data, given the study focus on adolescents with AIS undergoing spinal fusion surgery. The study design, with assessments once daily over 7 days per phase, yielded too few data points, further contributing to limited variation in data. This resulted in decreased power to detect statistically significant changes and/or correlations. Notably, a conservative alpha level was also used to avoid Type I error, yet increasing the risk of Type II error. Despite this, the results displayed both consistent and diverse patterns of psychological flexibility in adolescents after surgery and dynamic parent-adolescent processes of everyday life throughout post-surgical recovery for adolescents undergoing spinal fusion surgery. It is important to note that the study did not use an experimental design and that causal relationships cannot be assumed. Also, only one parent per adolescent was included, which should be taken into account when generalizing the results of the study.

## Conclusions and implications

In sum, the study results illustrated (i) the heterogeneity in the resilience factor psychological flexibility among adolescents after surgery, and (ii) the varying parent-adolescent dynamics of psychological mechanisms after surgery, across individuals and time. The findings imply the need for idiographic, process-based approaches in pain research. Diary methods are essential to capture the dynamics of the mechanisms at play in daily life, predicting a patients’ recovery process. Other mechanisms than psychological flexibility, such as anxiety or pain catastrophizing, in both adolescents and parents, and the interplay between psychological factors and daily pain intensity during the post-surgical recovery process, are also highly relevant research targets. Future studies should use more data points, perhaps twice daily or for longer time-periods. Time Series Analysis could be used for data collected at successive, equally spaced points in time to identify trends in the data. Dyadic Growth Curve Modelling could be used to examine how adolescents’ and parents’ individual trajectories of change are related over time. Clinically, the results highlight the need for individual assessments and flexible treatment application to actually address the risk and resilience factors at play for each particular patient at a given time. Timely individualized treatments would enhance pain treatment effectiveness in health care overall, and promote recovery for individual patients.

## Data Availability

The raw data supporting the conclusions of this article will be made available by the authors, without undue reservation.
